# Socioemotional Skills in the Teaching-Learning Process Mediated by Medium- and High-Fidelity Clinical Simulation in Nursing Students: Protocol for a Scoping Review

**DOI:** 10.2196/56436

**Published:** 2024-08-19

**Authors:** Luz Mery Contreras-Ramos, Elveny Laguado Jaimes, Nelly Esperanza Jaimes Carvajal, Marleny Pico Ferreira, Magda Liliana Villamizar-Osorio

**Affiliations:** 1 Faculty of Nursing Universidad Cooperativa de Colombia Bucaramanga Colombia

**Keywords:** social skills, nursing students, high-fidelity simulation training

## Abstract

**Background:**

In nursing education, contact with real scenarios implies the design of favorable experiences to develop prioritization, reasoning, critical thinking, and management skills that support future practice. In the context of the teaching-learning process, simulation emerges as a support strategy, but its use and management require the knowledge and appropriation of teachers. Clinical simulation during education promotes growth in technical skills and aptitudes such as critical thinking, emotional management, organization, delegation, and teamwork. The culmination positively impacts the student, reflecting on their confidence, security, and adaptability to unexpected or unknown situations and risks.

**Objective:**

The aim of this scoping review is to determine the socioemotional skills described during the teaching-learning process mediated by medium- and high-fidelity clinical simulation in nursing students.

**Methods:**

The main concepts and limits of the research area will be determined according to the 5 phases of a scoping review proposed by Arksey and O’Malley. Research articles and postgraduate theses published between 2010 and 2023 in English and Spanish will be considered. Dissertation-type documents, book chapters, editorials, abstracts, and articles focused on clinical simulation among nursing professionals will be excluded. The articles will be retrieved from databases available at the Universidad Cooperativa de Colombia, along with CINAHL, Scielo, and PubMed. The search strategy will be based on the Population-Concept-Context framework. Article selection will be carried out by 2 independent evaluators who will review titles and abstracts in stage 1 and the full text in stage 2. A database of retrieved articles will be built with the variables of interest. A qualitative thematic analysis will be conducted by 5 independent reviewers to provide an overview of the literature, focusing on identifying similarities and contrasts between studies and contributions related to the aspects of social skills described in nursing students.

**Results:**

The investigation has not yet started. The findings aim to focus on variables within the academic environment that, when correlated with the clinical simulation experience, may determine student learning. The working hypothesis is that students who experience greater satisfaction or possess better communication skills also demonstrate superior performance during high-fidelity simulation activities. The most relevant results will be contrasted considering the stated objective and knowledge gaps. Key aspects will also be compared with other reviews addressing related topics such as communication, self-efficacy, and self-confidence. Skills described by other authors that were not considered in the initial literature review will also be mentioned.

**Conclusions:**

Educational institutions are responsible for including learning experiences in controlled environments such as medium- and high-fidelity simulation to ensure the acquisition of technical capabilities and additional socioemotional skills. Recognizing and managing emotions is necessary to provide adequate care for users of health care services and for the increased effectiveness of professionals.

**Trial Registration:**

Open Science Framework p4ays; https://osf.io/p4ays

**International Registered Report Identifier (IRRID):**

PRR1-10.2196/56436

## Introduction

In the context of the teaching-learning process, simulation emerges as a support strategy; however, the use and management of simulators require the knowledge and appropriation of teachers [1]. Simulation is how a trained teacher creates scenarios to promote autonomous, collaborative learning along with critical and reflective thinking [2]. In turn, the mediator can formulate professional skills that respond to the needs of the context [3]. A requirement for this type of simulation is that teachers are trained in a pedagogical strategy for practice in the development of a model of enhancing competencies and clinical judgment in nursing [4].

Medium- and high-fidelity simulation has been used in nursing education. These scenarios reflect environments such as a hospital room, surgery room, or intensive care unit. Typically, a life-sized mannequin is used, which is connected to a computer system that simulates physiological variables such as vital signs, heart sounds, and respiratory sounds. The proposed cases can vary in complexity and aim for students to experience a situation similar to reality and acquire appropriate technical skills [5].

The implementation of this methodology among nursing students is supported by the use of technology for the training of facts and concepts, critical thinking, decision-making, and reasoning skills, which is then used to assess the progress of skills competencies and care interventions [6]. In this way, safe environments are generated that enable caring for a patient in a real situation, thus developing autonomous learning. Therefore, the main purpose of clinical simulation is to learn from significant experiences that allow building knowledge, representing a teaching-learning strategy that reflects the clinical practice environment, and finally offers the opportunity for evaluation of the training [7].

In nursing education, contact with real scenarios necessitates the design of favorable experiences to develop prioritization, reasoning, critical thinking, and management skills that support future practice [8]. Clinical simulation during education promotes technical skills and aptitudes, critical thinking, emotional management, organization, delegation, and teamwork, which has a positive impact on the student, reflecting on confidence, security, reaction to unexpected or unknown situations, and risk [9,10].

Barra and Calisto-Alegría [11] mentioned that simulation favors procedural skills through practice and repetition. They identified anxiety and shortcomings in the inputs, recommending methodological and didactic aspects in curricular proposals [11]. In addition, establishing socioemotional competencies of future nursing professionals will influence the quality of care, along with academic and effective performance via training, and improvement through clinical simulation is a favorable methodology for learning and evaluating socioemotional skills [12].

Understanding the patterns of communication in simulated situations in education is relevant as it generates trust in the clinical environment, which can in turn increase the efficiency of education in the processes of interaction with peers and patients [13].

Similarly, it is important to highlight the empathy that is required in nursing care, which is an art and the ethical center of the act of caring. The nurse is not exclusively limited to operational tasks, as nursing requires the establishment of interpersonal relationships with patients; therefore, communication and empathy are complementary to the knowledge and procedural skills necessary for an optimal condition of care. Through deliberate practice in simulation, one can effectively promote and improve the effectiveness of communication skills as well as the empathy and self-efficacy of students [14-16].

The aim of this study is to help identify the response patterns of teaching and learning methods or strategies. These patterns are manifested as emotional effects of students in a clinical simulation environment, which can cause tension, anxiety, and stress in students when facing nursing practices [17]. Toward this end, we will perform a scoping review to determine the socioemotional skills described during the teaching-learning process mediated by medium- and high-fidelity clinical simulation among nursing students.

## Methods

### Study Design and Registration

A scoping review will be carried out, which involves establishing the main concepts and their limits of a given area of research interest [18]. This study considers publications on the teaching-learning process for nursing in clinical simulation, high- and medium-fidelity simulation guides, and drug administration processes. The 5 phases proposed by Arksey and O’Malley [19] will be followed to carry out the review: (1) identifying the research question; (2) identifying relevant studies; (3) study selection; (4) charting the data; and (5) collating, summarizing, and reporting results.

This study represents the largest such investigation conducted as of January 2023 when the protocol was registered on the Open Science Framework under the identifier p4ays.

### Eligibility Criteria

Full-text articles published on the central theme of the study, including postgraduate theses, studies using different quantitative methodologies, mixed methods studies, and systematic reviews, will be eligible for inclusion in the review. Articles published from 2010 to 2024 involving nursing student populations will be considered eligible. In addition, articles written in English and Spanish are considered. Dissertation-type documents, book chapters, editorials, abstracts, and articles focused on clinical simulation in nursing professionals will be excluded.

### Information Sources

The databases available at the Universidad Cooperativa de Colombia (OVID Medicina y Enfermería, Science Direct, Sage Journals, Proquest Psychology Database, Proquest Social Science Database, Proquest UK & Ireland Database, Psycarticles, Redalyc, and Dialnet) and databases such as CINAHL, Scielo, and PubMed will be searched for relevant articles.

### Search Strategy

The Population (or Participants)-Concept-Context framework recommended by the Joanna Briggs Institute [19] will be followed to identify the main concepts to orient the search strategy, which are summarized in Textbox 1.

**Population-Concept-Context framework of the study.**

**Population**

Nursing students

**Concept**

The personal set of abilities required to successfully interact and communicate with others, both verbally and nonverbally through gestures, body language, and personal appearance

**Context**

The teaching-learning process mediated by medium- and high-fidelity clinical simulation

To achieve standardization of search terms, the article’s keywords will be selected from the Descritores em Ciências da Saúde (DeCS) website for the descriptors in Spanish [20] and from Medical Subject Headings (MeSH) for the descriptors in English [21], which are presented in Table 1.

**Table 1:** Standardized search terms.

Spanish terms	English terms	Definition
Enseñanza mediante simulación de alta fidelidad	High-fidelity simulation training	A controlled learning environment that closely represents reality
Estudiantes de enfermería (synonyms: alumnos de enfermería, estudiante de enfermería)	Nursing students (synonyms: pupil nurses, pupil nurse)	Individuals enrolled in a school of nursing or a formal educational program leading to a degree in nursing
Entrenamiento simulado	Simulation training (synonym: interactive learning)	A highly customized interactive medium or program that allows individuals to learn and practice real-world activities in an accurate, realistic, safe, and secure environment
Modelos educacionales comunicación en salud	Educational model (synonyms: instructional models, instructional model, educational model)	Theoretical models that propose methods of learning or teaching as a basis or adjunct to changes in attitude or behavior. These educational interventions are usually applied in the fields of health and patient education but are not restricted to patient care
Comunicación en salud (synonyms: información y comunicación en salud, información y comunicación en la salud)	Health communication (synonym: health communications)	The transfer of information from experts in the medical and public health fields to patients and the public; the study and use of communication strategies to inform and influence individual and community decisions that enhance health
Habilidades sociales (synonym: habilidades socioemocionales)	Social skill (synonyms: social skills, social abilities, social ability, interpersonal skills, interpersonal skill, social competence)	The personal set of abilities required to successfully interact and communicate with others, both verbally and nonverbally through gestures, body language, and personal appearance

### Selection of Sources of Evidence

The search will begin in the databases available in the university library and then the literature review will be expanded to other open access databases. The compilation will be carried out in two stages.

The first stage involves title and abstract selection, which will be carried out by 2 reviewers independently considering the inclusion exclusion criteria. In this first stage, the selected keywords will be put to the test to verify that the articles that respond to the proposed theme are adequately recovered with these terms.

In the second stage, the full text of the articles selected in the first phase will be read by 2 reviewers independently and then the results obtained will be contrasted. Any disagreement between the 2 reviewers will be resolved by a third reviewer.

### Data Charting Process

The extracted data will be organized in a Microsoft Excel spreadsheet, including the following aspects: title, bibliographic source, objective of the study, country of origin, type of study, design, evaluated social skills, simulation scenario, and level of the students.

Subsequently, a qualitative thematic analysis will be performed by 5 independent reviewers to offer an overview of the literature with the aim of identifying similarities, contrasts among authors, and contributions in the aspects of social skills described in nursing students. Because this is a scoping review, it will encompass many types of evidence and therefore it is not considered pertinent to apply bias control to the sources of information or the results of individual studies [22].

### Synthesis of Results

The characteristics of the selected articles related to socioemotional skills mediated by clinical simulation as part of the teaching-learning process for nursing education will be described in detail. The data will be presented using summary tables and grouped figures.

### Ethical Considerations

In accordance with current international regulations [23,24], this project does not require approval from an ethics committee, as the research does not involve the collection of new data from humans, does not present any risks to participants, and is based on the analysis of secondary data from previously published research.

## Results

### Selection of Sources of Evidence

**Figure 1:**
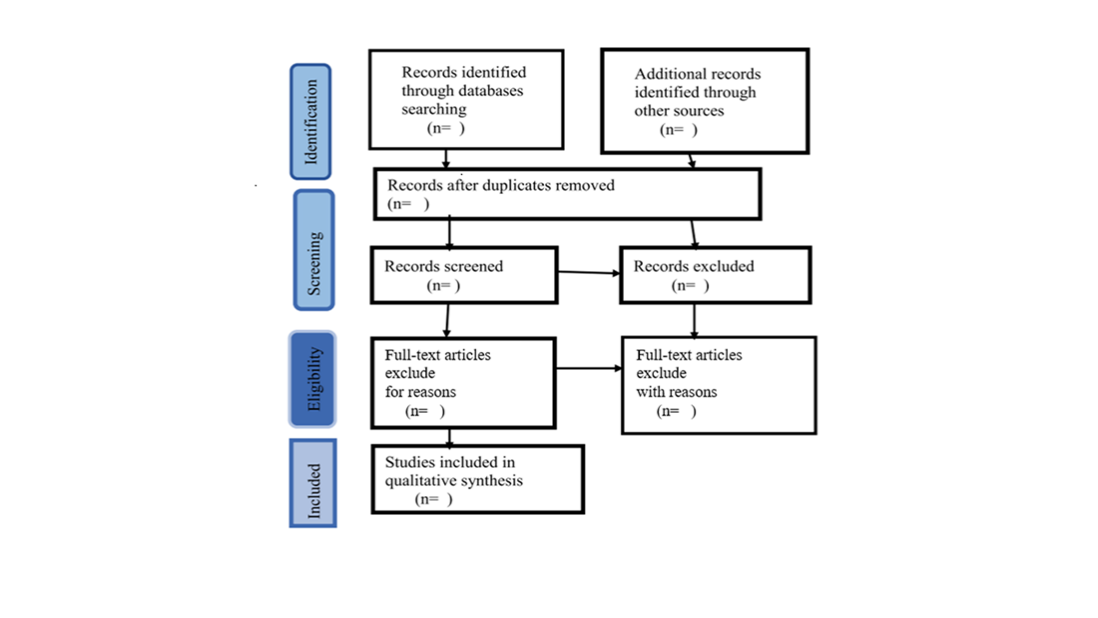
Example PRISMA-ScR (Preferred Reporting Items for Systematic Reviews and Meta-Analyses extension for Scoping Reviews) flowchart for study inclusion.

The article selection process will be summarized using a PRISMA-ScR (Preferred Reporting Items for Systematic Reviews and Meta-Analyses extension for Scoping Reviews) flowchart [22], as shown in Figure 1.

### Characteristics of Sources of Evidence

A table will be developed to summarize the main characteristics of the selected articles for the research objective, including the country where the study was conducted, design, and identified communication skills. The findings of this scoping review aim to focus on variables within the academic environment that, when related with the clinical simulation experience, may determine the outcomes of student learning. The working hypothesis posited is that students who experience greater satisfaction or possess better communication skills will also demonstrate superior performance during high-fidelity simulation activities.

### Synthesis of Results

We will use a narrative approach for data extraction, including descriptions of author characteristics, year of publication, study design, objective, sample size, country, level of education, type of clinical simulation, and categorization by themes. The purpose is to consolidate knowledge about the study phenomenon. Toward this end, the 5 reviewers will apply criteria for critical reading to evaluate the evidence [25]. In case of any disagreement regarding theme categorization, resolution will be achieved through consensus among the reviewers.

## Discussion

### Summary of Evidence

Medium- and high-fidelity clinical simulation is a learning tool for technical capabilities and generates response patterns in students that can influence the acquisition of the socioemotional skills necessary for the adequate performance of nursing students in the real environment [26]. Social skills influence the academic and clinical performance of the student in training, complementing preparation for real-world scenarios.

We predict identifying a relationship between exposure to clinical simulation and the improvement of skills such as communication, self-confidence, and problem-solving, along with an increase in satisfaction perception.

The most relevant results will be contrasted considering the stated objective and knowledge gaps. Key aspects will also be compared with other reviews addressing related topics such as communication, self-efficacy, and self-confidence. Skills described by other authors that were not considered in the initial literature review will also be mentioned.

### Limitations

Limitations related to the omission of gray literature or documents written in languages other than Spanish and English will be discussed, and recommendations for conducting future research will be proposed.

The main limitation is the lack of evaluation of the quality of the articles that will be selected; however, Arskey and O’Malley [18] specify that a scoping review does not aim to assess the quality of the evidence but rather to answer the research question. Additionally, there are limited studies on medium- and high-fidelity clinical simulation in nursing students available to date.

### Conclusions

In accordance with the findings, the most significant results will be outlined. Providing adequate nursing care involves knowledge of equipment, advanced health care practices, and advanced technical capabilities. Educational institutions are responsible for including learning experiences in controlled environments such as medium- and high-fidelity simulation to ensure the acquisition of technical capabilities as well as socioemotional skills. Recognizing and managing emotions is necessary to provide adequate care for users of health care services and for the increased effectiveness of professionals.

## Abbreviations

DeCS: Descriptores en Ciencias de la Salud
MeSH: Medical Subject Headings
PRISMA-ScR: Preferred Reporting Items for Systematic Reviews and Meta-Analyses extension for Scoping Reviews
